# Multifamily Group Psychoeducation and Cognitive Remediation for First-Episode Psychosis: A Randomized Controlled Trial

**DOI:** 10.1186/1471-244X-11-9

**Published:** 2011-01-12

**Authors:** Nicholas JK Breitborde, Francisco A Moreno, Natalie Mai-Dixon, Rachele Peterson, Linda Durst, Beth Bernstein, Seenaiah Byreddy, William R McFarlane

**Affiliations:** 1 Department of Psychiatry, University of Arizona, 1501 N. Campbell Ave., PO Box 245002, Tucson, AZ, 85724-5002, USA; 2 Department of Psychiatry, University Physicians Hospital, 2800 E. Ajo Way, Tucson, Arizona, 85713, USA; 3 Department of Psychiatry, University of Rochester Medical Center, 601 Elmwood Ave., Rochester, New York, 14642, USA; 4 Department of Psychiatry, Maine Medical Center, 295 Park Ave., Portland, Maine, 04102, USA

## Abstract

**Background:**

Multifamily group psychoeducation (MFG) has been shown to reduce relapse rates among individuals with first-episode psychosis. However, given the cognitive demands associated with participating in this intervention (e.g., learning and applying a structured problem-solving activity), the cognitive deficits that accompany psychotic disorders may limit the ability of certain individuals to benefit from this intervention. Thus, the goal of this study is to examine whether individuals with first-episode psychosis who participate simultaneously in MFG and cognitive remediation--an intervention shown to improve cognitive functioning among individuals with psychotic disorders--will be less likely to experience a relapse than individuals who participate in MFG alone.

**Methods/Design:**

Forty individuals with first-episode psychosis and their caregiving relative will be recruited to participate in this study. Individuals with first-episode psychosis will be randomized to one of two conditions: (i) MFG with concurrent participation in cognitive remediation or (ii) MFG alone. The primary outcome for this study is relapse of psychotic symptoms. We will also examine secondary outcomes among both individuals with first-episode psychosis (i.e., social and vocational functioning, health-related quality of life, service utilization, independent living status, and cognitive functioning) and their caregiving relatives (i.e., caregiver burden, anxiety, and depression)

**Discussion:**

Cognitive remediation offers the possibility of ameliorating a specific deficit (i.e., deficits in cognitive functioning) that often accompanies psychotic symptoms and may restrict the magnitude of the clinical benefits derived from MFG.

**Trial Registration:**

ClinicalTrials (NCT): NCT01196286

## Background

There is growing evidence that the majority of the psychosocial deterioration that accompanies psychotic disorders occurs during the first few years of illness [1-3] and that the prevention or delay of early deterioration may be associated with a better course of illness [4-7]. One intervention which has been shown to be particularly effective in the treatment of psychotic disorders is family psychoeducation--an umbrella term for a group of interventions that provide families with education about psychotic disorders and strategies to improve problem-solving skills and communication within the family [8]. To date, multiple studies have demonstrated that the receipt of family psychoeducation is associated with lower rates of relapse among individuals with psychotic disorders [9,10] with individuals with first-episode psychosis experiencing greater clinical benefits than individuals later in the course of a psychotic disorder [11,12].

One particular form of family psychoeducation which has shown promise among individuals with first-episode psychosis is multifamily group psychoeducation (MFG) [11]. This intervention provides participants with information about the course and treatment of psychotic disorders and trains participants in the use of a structured problem-solving exercise designed to help them navigate the many challenges associated with living with a psychotic disorder or caring for a relative with a psychotic disorder. Among individuals with psychotic disorders, participation in MFG is associated with reduced rates of relapse [13,14], and the clinical benefit of this intervention appears to be greater among individuals with first-episode psychosis as opposed to individuals with a chronic psychotic disorder [11]. The success of this intervention among individuals with first-episode psychosis has led to the incorporation of MFG within several major international studies of first-episode psychosis (e.g., OPUS [15] and TIPS [16]).

However, like all psychosocial interventions, some individuals who participate in MFG will still experience negative health outcomes. With regard to individuals with first-episode psychosis, approximately 20% may experience a symptomatic relapse and 50% may be hospitalized over a two-year period despite participating in family psychoeducation [11,13]. Thus, despite the clear clinical benefits associated with participation in MFG, there is still room for improvement with regard to the clinical outcomes of individuals who participate in this intervention.

One factor that may limit the benefit of psychosocial treatments (e.g., MFG) for psychosis is the cognitive deficits that tend to accompany psychotic disorders [17,18]. Cognitive deficits in areas such as problem-solving ability, verbal memory, and attention are common in individuals with psychotic disorders [19,20] (including those early in the course of a psychotic disorder [21,22]) and have been recognized as a "rate-limiting" factor which may hinder individuals' ability to learn and execute new skills [18,23]. In the context of MFG, these cognitive deficits may hinder an individual's ability to learn and participate in the problem-solving activity which is the hallmark of MFG. Addressing these cognitive deficits, in particular those related to problem-solving, could potentially facilitate greater participation and understanding of the MFG problem-solving activity among individuals with first-episode psychosis--thereby facilitating greater clinical benefits associated with participation in this intervention.

Recently, greater attention has been directed toward the development of strategies to ameliorate the cognitive deficits that accompany psychotic disorders. One strategy which has been shown to be successful in this endeavor is cognitive remediation (CR). This intervention, which is recognized as a "best practice" in the treatment of psychotic disorders [24,25], is typically comprised of a series of repeated exercises delivered by a clinician or via a computer that are designed to improve performance in cognitive functioning. A recent-meta-analysis has shown that participation in cognitive remediation programs is associated with improvements in multiple domains of cognitive functioning, including problem-solving ability [26]. The success of CR in improving problem-solving skills (and other areas of cognitive functioning) raises the possibility that individuals with first-episode psychosis who participate concurrently in MFG and CR may be better able to learn and apply the problem-solving activity completed during MFG sessions. This, in turn, could lead to improvements in outcomes experienced by these individuals.

Thus, the goal of this study is to examine whether concurrent participation in MFG and CR is associated with better outcomes among individuals with first-episode psychosis than participation in MFG alone. We hypothesize that relapse rates will be lower among individuals who participate in the MFG and CR condition as opposed to MFG alone. However, recognizing that the benefits of MFG and CR may not be limited to relapse alone, we will also examine the benefits of these interventions with regard to secondary outcome measures for both individuals with first-episode psychosis and their caregiving relatives.

## Methods/Design

This project was approved University of Arizona Human Subjects Protection Program.

### Participants

#### Sample Characteristics

Individuals with first-episode psychosis and their caregiving relatives will be recruited from the Early Psychosis Intervention Center (EPICENTER) at University Physicians Hospital. EPICENTER is an outpatient treatment program that provides evidence-based psychosocial treatments for individuals experiencing their first psychotic episode. Inclusion criteria for participants at EPICENTER are (i) a diagnosis of an affective or schizophrenia spectrum psychotic disorder as determined by the Structured Clinical Interview for the DSM-IV (SCID [27]), (ii) less than 5 years of frank psychotic symptoms as determined by the Symptom Onset in Schizophrenia inventory (SOS [28]), (iii) being between the ages of 18-35, and (iv) willingness to receive treatment at EPICENTER. The durational criteria for psychotic symptoms (< 5 years) is based on the operational definition of first-episode psychosis outlined by Breitborde and colleagues [29]. Individuals with first-episode psychosis are excluded from EPICENTER if they meet criteria for substance-induced psychosis as determined by the SCID, are unwilling or unable to provide informed consent, or meet criteria for a diagnosis of mental retardation. Caregiving relatives are defined as someone with whom the individual with first-episode psychosis maintains considerable face-to-face contact (≥ 10 hours per week). Family caregivers do not need to be biological relatives of the individual with first-episode psychosis. It is anticipated that some individuals with first-episode psychosis will have more than one caregiving relative who wishes to participate in the study; hence, we anticipate recruiting ≈1.5 familial caregivers for each individual with first-episode psychosis.

Given that the onset of psychosis typically occurs between the ages of 15-35 [median ≈ 22-23 years] [30], we expect that our cohort of individuals with recent-onset psychosis will comprised largely of young adults. As noted earlier, due to EPICENTER inclusion criteria, no individuals younger than 18 years old will be included in this study. As the prevalence of psychotic disorders within the United States does not appear to differ across racial or ethnic groups [31], we expect that racial and ethnic distribution of individuals with first-episode psychosis who participate in this study will be consistent with the racial and ethnic distribution of Tucson, Arizona. Per the 2000 U.S. Census data for Tucson, Arizona, this would lead us to expect that the racial distribution of our sample will be 70% White, 4% African American, 2% American Indian, 2% Asian American, <1% Native Hawaiian or other Pacific Islander, 4% multiracial, and 17% other. With regard to ethnicity, we expect that the overall sample will be comprised of 36% Hispanic/Latino individuals and 64% non-Hispanic/Latino individuals. We expect to find a similar ethnic and racial breakdown among the family caregivers who participate in this study.

First-episode psychosis studies have long reported recruiting a preponderance of male subjects [32]. Thus, we expect that our sample of individuals with first-episode psychosis will be largely male (≈70%). Conversely, studies of family caregivers of individuals with psychotic disorders have historically recruited a preponderance of female caregivers [33]. As such, we expect that our sample of caregivers will be largely female (≈70%).

#### Number of Participants and Power Analysis

Current recommendations for a priori determination of the number of subjects to include in a study suggest the inclusion of sufficient subjects to maintain adequate statistical power to detect a clinically meaningful effect size [34]. One such measure, Number Needed to Treat (NNT) [35], has been identified as particularly useful in conveying clinical significance and in guiding the design of randomized clinical trials [36]. NNT provides an estimate of the number of individuals who would need to receive a treatment in order to prevent the occurrence of one negative outcome. With regard to family psychoeducation, a recent meta-analysis found that the NTT for this intervention was 8; (95% CI 6-18) [9]. This suggests that this intervention would need to be provided to 8 individuals to prevent one relapse. Although there is no established criteria for a clinically meaningful reduction in NNT [36], for the current study we defined a clinically meaningful benefit of the MFG and CR condition as an NNT one-half the size of the NNT for MFG along (i.e., an NNT for MFG and CR = 4). This value (i.e., NNT = 4) falls outside of the 95% confidence interval of the NNT for family psychoeducation alone as reported in a past meta-analysis [9] and is consistent with the NNT value use to determine a priori statistical power for most randomized controlled trials of interventions for mental illnesses [36]. Using these NNT values and the pwr software package [37] developed for the R statistical platform [38], we determined that 17 families (i.e., individual with first-episode psychosis and caregiving relative[s]) would need to be allocated to both the MFG-CR and MFG alone conditions, respectively, to ensure statistical power of 0.80 (i.e., total sample size = 34). To protect against subject attrition, we will recruit an additional 6 families (i.e., ≈20% of the total sample size), bringing the total sample size to 40.

### Randomization and Treatment Allocation

**Figure 1:**
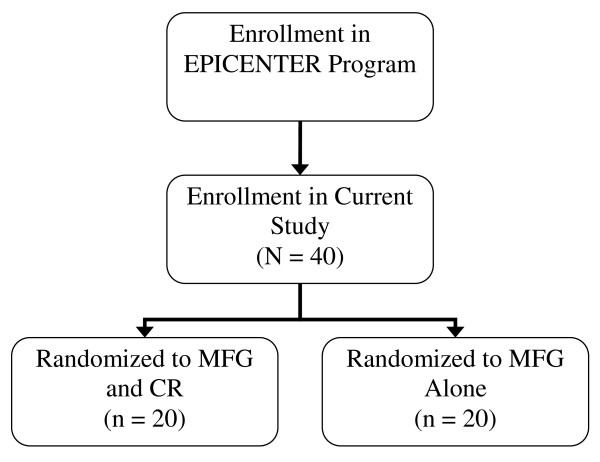
Patient Flow Diagram

Treatment allocation for this study is depicted in Figure 1. Upon enrollment in the project, individuals with first-episode psychosis will be randomized to either the MFG and CR condition or the MFG alone condition. Randomization will be completed using a block randomization procedure with blocks of varying sizes.

### Interventions

#### Multifamily Group Psychoeducation

Per the protocol outlined by McFarlane [11], the MFG intervention involves three phases: (i) joining, a process of engaging patients and their key family members, (ii) a psychoeducational workshop, and (iii) multifamily problem-solving sessions. During the joining phase, family members meet with the clinician who will lead the MFG to discuss their ill relative's clinical history, the family's experience and understanding of their ill relative's illness, and family members' concerns and questions with regard to participating in a multifamily group. Concurrent to these sessions with the family, the individual with first-episode psychosis will also complete three individual sessions with the clinician to build rapport and trust in the relationship between the clinician and the individual with first-episode psychosis. Following the completion of the joining phase, family members and clinically stable patients participate in a day-long educational workshop on psychosis which provides an overview of the causes and prognosis of psychotic disorders, current treatments for these disorders, and the ways in which family members may be affected by severe mental illness in the family. Family members are also presented with guidelines for illness management as well as strategies to maintain family balance and well-being. Following the completion of the psychoeducational workshop, families and their ill relatives begin to participate in bi-weekly multifamily problem-solving sessions. During the problem solving sessions, caregivers and ill relatives identify challenges or problems occurring in their lives and identify possible solutions to these problems through a structured problem-solving activity.

All individuals with first-episode psychosis will participate in the MFG intervention for twelve months. This duration of treatment is consistent with recommendations from the Patient Outcomes Research Team (PORT) convened by the Agency for Health Care Policy and Research and the National Institute of Mental Health [39,40]. Of note, unlike the traditional MFG model, family groups in this study will be run using rolling admissions with families graduating from the group after twelve months of participation.

#### Cognitive Remediation

Individuals with first-episode psychosis who are randomized to the MFG and CR condition will complete the cognitive remediation program PSSCogRehab [41]. This computerized cognitive remediation program provides participants with training in 4 areas of cognitive functioning: attention, visual-spatial abilities, memory, and problem-solving abilities. Participants initially complete simple tasks in each domain and, once mastered, gradually progress to more difficult tasks. Completion of the training program occurs once subjects have mastered all of the training tasks. This program has been frequently used in past studies of cognitive remediation in psychotic disorders [42-48], and more recently has been applied specifically among individuals early in the course of a psychotic illness [49,50]. This intervention has been shown to promote improvements in problem-solving among individuals with psychotic disorders [42], and has been administered successfully with other concurrent psychosocial interventions [44].

### Primary Outcome Measure

#### Relapse

Symptomatology among individuals with first-episode psychosis will be assessed using the Positive and Negative Syndrome Scale (PANSS) [51] on a weekly basis during their participation in the study. Based on participants' scores on this measure, the occurrence of a relapse will be determined using the criteria established by Nuechterlein and colleagues [52]. Of note, although the criteria outlined by Nuechterlein and colleagues were designed for use with the Brief Psychiatric Rating Scale (BPRS [53]), the specific items on the BPRS used to determine the occurrence of a relapse using the Nuechterlein criteria (i.e., hallucinations, unusual thought content, and conceptual disorganization) are also included in the PANSS (i.e., hallucinations, delusions, and conceptual disorganization). These shared items are scored in an identical manner on both measures and each item on BPRS has been shown to be strongly correlated with its comparable item on the PANSS (weighed kappas of 0.65 [good] to 0.86 [excellent]) [54].

### Secondary Outcome Measures

Recognizing that recovery from psychotic disorders involves more than just a remission of psychotic symptoms [55], we will also explore the benefit of combining MFG and CR on other outcomes among individuals with first-episode psychosis. These will include social and vocational functioning (Social Functioning Scale: SFS [56]), everyday functioning (brief form of the UCSD Performance-Based Skills Assessment: UPSA [57]), health-related quality of life (RAND 36-Item Health Survey [58]), service utilization (Service Utilization and Resources Form for Schizophrenia: SURF [59]), and independent living status. Independent living status will be assessed using the methodology outlined by Palmer et al. [60]. Per this methodology, subjects' living status will be rated on a 4-point scale ranging from (1) 'totally dependent' (i.e., living in a facility with 24-hour clinical care) to (4) 'independent' (i.e., living alone or with a partner who provides a level of support consistent in typical cohabitation relationships). These measures will be administered when subjects enroll in the study and again after the completion of 12 months of MFG.

Additionally, to replicate findings linking participation in CR to improved cognitive functioning among individuals with psychotic disorders [26], individuals with first-episode will complete the consensus cognitive battery developed by the National Institute of Mental Health's Measurement and Treatment Research to Improve Cognition in Schizophrenia (MATRICS) initiative [61]. Of note, this battery does include a specific assessment of problem-solving skills (i.e., the mazes subtest from the Neuropsychological Assessment Battery [62]). Participants in the MFG and CR condition will complete the MATRICS battery three times over the course of the study: (i) at enrollment; (ii) upon completion of CR intervention, and (iii) upon completion of 12 months of the MFG intervention. Individuals randomized to the MFG alone condition will complete the MATRICS battery three times over the course of the study: (i) at enrollment, (ii) at 10 weeks, and (iii) upon completion of 12 months of the MFG intervention

Caregiving relatives of individuals with psychotic disorders have also been shown to experience a reduction in caregiver burden and psychological distress (e.g., depression and anxiety) after participation in family psychoeducation [63,64]. Thus, we plan to conduct additional secondary analyses to examine whether caregivers whose ill relatives are in the MFG and CR group experience greater benefits in these areas as compared to caregivers whose ill relatives are in the MFG alone condition. Caregiver burden will be assessed using the Burden Assessment Scale [BAS] [65], and depression and anxiety will be assessed using the Beck Depression Inventory [BDI] [66] and Beck Anxiety Inventory [BAI] [67], respectively. These measures will be administered upon enrollment to the study and after completion of 12 months of MFG.

### Proposed Analyses

All analyses will be completed using an "intention-to-treat" principle [68] such that data from all subjects will be included in the analysis regardless of their level of adherence to the interventions over the course of the study.

The association between intervention condition (i.e., MFG and CR vs. MFG alone) and relapse will be examined using a chi-square. However, in situations in which the requirements for this analysis are violated (e.g., expected value of any cell ≤ 5), Fisher's exact probability test [69] with the continuity correction proposed by Overall [70] will be used instead.

Per the recommendations outlined by Vickers and Altman [71], the association between intervention condition (i.e., MFG and CR vs. MFG alone) and continuous secondary outcome measures (e.g., caregiver burden and social functioning scores) will be examined using an analysis of covariance with participants' baseline scores on the secondary outcome measure included as a covariate. With regard to the association between intervention condition and categorical secondary outcome measures (e.g., employed vs. unemployed), a chi-square analysis will be used. However, in situations in which the requirements for this analysis are violated, Fisher's exact probability test [69] with the continuity correction proposed by Overall [70] will be used instead.

## Discussion

Multifamily group psychoeducation is an evidence-based and cost-effective treatment for psychotic disorders [13,14,72]. However, like all psychosocial interventions, certain individuals who participate in MFG will still go on to experience negative health outcomes. Cognitive remediation offers the possibility of ameliorating a specific deficit (i.e., a deficit in cognitive functioning) that often accompanies psychotic symptoms and may restrict the magnitude of the clinical benefits derived from MFG.

## Competing interests

The authors declare that they have no competing interests.

## Authors' contributions

Study concept and design: NJKB; Protocol management: NM-D, RP; Drafting of the manuscript: NJKB; Critical Revision of the manuscript: FAM, NM-D, RP, SB, WM. All authors approved the final version of this manuscript.

## Pre-publication history

The pre-publication history for this paper can be accessed here:

http://www.biomedcentral.com/1471-244X/11/9/prepub
